# Real-time inference of the end of an outbreak: Temporally aggregated disease incidence data and under-reporting

**DOI:** 10.1016/j.idm.2025.03.009

**Published:** 2025-04-01

**Authors:** I. Ogi-Gittins, J. Polonsky, M. Keita, S. Ahuka-Mundeke, W.S. Hart, M.J. Plank, B. Lambert, E.M. Hill, R.N. Thompson

**Affiliations:** a Mathematics Institute, University of Warwick, Coventry, UK; b Zeeman Institute for Systems Biology and Infectious Disease Epidemiology Research (SBIDER), University of Warwick, Coventry, UK; c Geneva Centre of Humanitarian Studies, University of Geneva, Geneva, Switzerland; d World Health Organization, Regional Office for Africa, Brazzaville, Republic of the Congo; e Institute of Global Health, Faculty of Medicine, University of Geneva, Geneva, Switzerland; f National Institute of Biomedical Research, Kinshasa, Democratic Republic of the Congo; g Mathematical Institute, University of Oxford, Oxford, UK; h School of Mathematics and Statistics, University of Canterbury, Christchurch, New Zealand; i Department of Statistics, University of Oxford, Oxford, UK; j Pandemic Sciences Institute, University of Oxford, Oxford, UK; k Civic Health Innovation Labs and Institute of Population Health, University of Liverpool, Liverpool, UK; l NIHR Health Protection Research Unit in Gastrointestinal Infections, University of Liverpool, Liverpool, UK

**Keywords:** Ebola virus disease, Interventions, End-of-outbreak probability, Renewal equation, Epidemic modelling

## Abstract

Professor Pierre Magal made important contributions to the field of mathematical biology before his death on February 20, 2024, including research in which epidemiological models were used to study the ends of infectious disease outbreaks. In related work, there has been interest in inferring (in real-time) when outbreaks have ended and control interventions can be relaxed. Here, we analyse data from the 2018 Ebola outbreak in Équateur Province, Democratic Republic of the Congo, during which an Ebola Response Team (ERT) was deployed to implement public health measures. We use a renewal equation transmission model to perform a *quasi* real-time investigation into when the ERT could be withdrawn safely at the tail end of the outbreak. Specifically, each week following the arrival of the ERT, we calculate the probability of future cases if the ERT is withdrawn. First, we show that similar estimates of the probability of future cases can be obtained from either daily or weekly case reports. This demonstrates that high temporal resolution case reporting may not always be necessary to determine when interventions can be relaxed. Second, we demonstrate how case under-reporting can be accounted for rigorously when estimating the probability of future cases. We find that, the lower the level of case reporting, the longer it is necessary to wait after the apparent final case before interventions can be removed safely (with only a small probability of additional cases). Finally, we show how uncertainty in the extent of case reporting can be included in estimates of the probability of future cases. Our research highlights the importance of accounting for under-reporting in deciding when to remove interventions at the tail ends of infectious disease outbreaks.

## 1 Introduction

Early in the COVID-19 pandemic, Professor Pierre Magal and colleagues (including the final author of this article) had a keen interest in using epidemiological models to infer when outbreak waves would end (Griette et al., 2022). A key finding of their research was that the assumed level of case reporting affects predictions about when localised outbreaks will finish.

In related studies, quantitative frameworks have been developed for determining when an outbreak is over (Linton et al., 2022; Nishiura, Miyamatsu, & Mizumoto, 2016). Rather than attempting to predict the end of an outbreak in advance, the main focus of that work has been real-time estimation of the end-of-outbreak probability (i.e., the probability that no future cases will occur in the current outbreak) (Bradbury et al., 2023; Hart et al., 2024). Equivalently, the probability of future cases occurring can be estimated (this quantity is one minus the end-of-outbreak probability) (Nishiura, Miyamatsu, & Mizumoto, 2016). An outbreak can theoretically be declared over, and interventions relaxed or withdrawn, when the probability of future cases falls below a pre-specified value that reflects policy makers’ willingness to accept a risk of further transmission (Thompson et al., 2024).

Epidemiological assessments during outbreaks are often affected by the frequency of data reporting. For example, when pathogen transmissibility is inferred from case data, more sophisticated methods may be required when disease incidence time series data are aggregated weekly than when daily case numbers are reported (Nash et al., 2022, 2023; Ogi-Gittins et al., 2024, 2025). In addition, epidemiological analyses may be less reliable in the presence of case under-reporting (Atkins et al., 2015; Dalziel et al., 2018; Gamado et al., 2014; Gignoux et al., 2015). Unfortunately, both temporal aggregation of case reports into weekly values and under-reporting are commonplace during outbreaks of many pathogens (Centers for Disease Control and Prevention, 2024; Gibbons et al., 2014), with under-reporting being particularly likely in the low-resource settings in which high-threat pathogens frequently emerge and spread extensively.

**Fig. 1:**
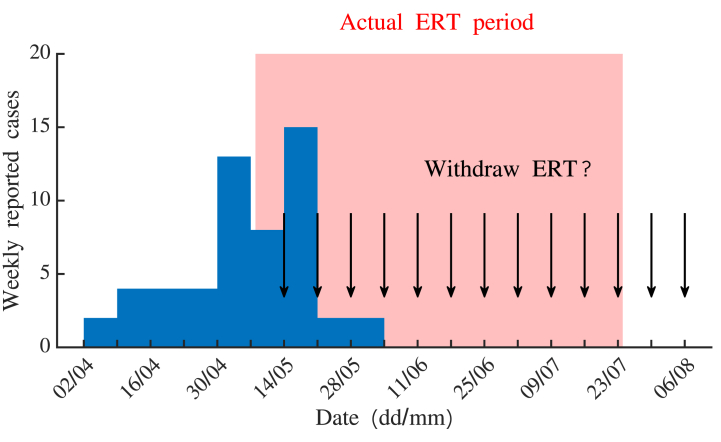
**Weekly numbers of reported cases in the 2018 EVD outbreak in Équateur Province, DRC.** The ERT was deployed from 8th May to 24th July (red shaded region). In our analyses, we consider estimating the probability of future cases at the beginning of each epidemiological week following the arrival of the ERT (black arrows).

In this article, we build on recent developments in estimation of end-of-outbreak probabilities and explore how temporal aggregation of epidemiological data and under-reporting affect quantitative assessments of when outbreaks are over. Specifically, we consider an outbreak of Ebola virus disease (EVD) that occurred in Équateur Province, Democratic Republic of the Congo (DRC), in 2018. In the outbreak, 54 cases were recorded, and both daily and weekly aggregated case reports are now available (Fig. 1 and Fig. S1). The Ebola Response Team (ERT), co-ordinated by the World Health Organization (WHO) and DRC Ministry of Health, was deployed to implement public health measures to contain the outbreak.

First, we apply a previously published method (Thompson et al., 2024) to calculate the risk of withdrawing the ERT each week, as quantified by the probability of future cases occurring if the ERT is withdrawn (under the assumption that pathogen transmissibility reverts to its inferred pre-ERT value as soon as the ERT is withdrawn). We show that the estimated probability of future cases is very similar if daily or weekly case numbers are used. This indicates that quantitative methods for determining when public health measures can be removed or relaxed at the end of an EVD outbreak can be robust when only weekly disease incidence data are reported, rather than daily data. Having verified that reliable estimates of the probability of future cases can be obtained from the weekly disease incidence time series, we show how case under-reporting can be accounted for (using Gibbs sampling) when estimating the probability of future cases. Our main finding is that a greater extent of case under-reporting requires policy makers to wait longer before interventions can be withdrawn with confidence. As we show, our method also permits uncertainty in the level of case reporting to be accounted for, and we perform supplementary analyses in which we verify the robustness of our main finding to our modelling assumptions (for example, we consider a scenario in which there is a delay following the deployment of the ERT before it becomes effective). The modelling framework presented here enables estimates of the probability of future cases to be obtained in real-time during outbreaks, while accounting rigorously for the possibility of case under-reporting, as a guide for public health decision making.

## 2 Methods

**Fig. 2:**
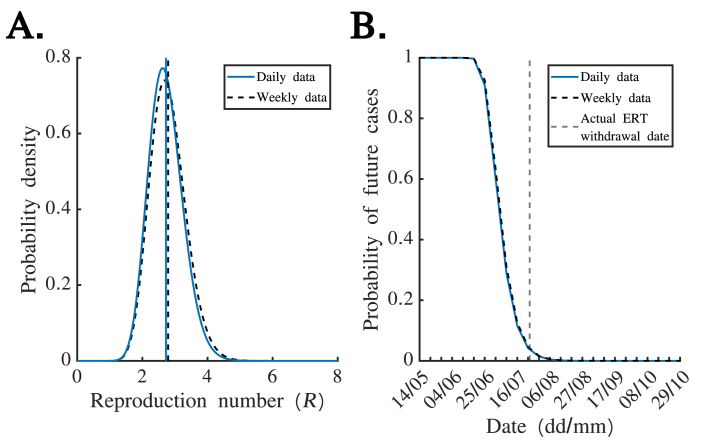
**Probability of future cases estimated from either weekly or daily disease incidence time series data, assuming perfect case reporting.** A. Posterior estimate of the reproduction number in the absence of the ERT (R), estimated using either the weekly disease incidence data (black dashed; see section 2.2 for further details) or the daily disease incidence data (blue). The mean estimates of R are denoted by the corresponding vertical lines. B. *Quasi* real-time estimates of the probability of future cases, obtained using either the weekly disease incidence data (black dashed; equation (2)) or the daily disease incidence data (blue). The actual ERT withdrawal date is indicated using a grey dashed vertical line.

Here, we describe the methods underlying our analyses. Throughout this section, we assume that calendar time (t) is measured in weeks, since weekly disease incidence data were made publicly available during the 2018 EVD outbreak in Équateur Province, DRC. However, in Fig. 2, we also analyse the daily disease incidence data underlying the weekly reported values (Fig. S1; also shown in Fig. S3A of the previous study by Thompson et al. (Thompson et al., 2024)). In that scenario, the methods described here were applied in the same way, but instead using a timescale of days.

### 2.1 Epidemiological data

We consider a disease incidence time series dataset from the 2018 EVD outbreak in Équateur Province, DRC. In total, 54 cases (40 confirmed cases and 14 probable cases) occurred between 5th April and 2nd June (inclusive). The ERT was deployed on 8th May and was withdrawn on 24th July. The ERT withdrawal date was based on the general (i.e., not outbreak-specific) WHO guideline to declare an EVD outbreak over and relax associated interventions after a period of 42 case-free days following the recovery or burial of the apparent final case (WHO, 2020); the rationale for this criterion is described in the Discussion. The overall goal of our analysis is to determine the risk of withdrawing the ERT (i.e., the probability of future cases) at the beginning of each week following the deployment of the ERT, as could have been estimated for this specific EVD outbreak (Fig. 1).

### 2.2 Inference of pathogen transmissibility

When the ERT is not in place, we assume that the number of cases in any week t, $I_{t}$, is drawn from a Poisson distribution with mean

$$E\left(I_{t}|{\left\{I_{k}\right\}}_{k=1}^{t-1},R,w\right)=R\sum_{s=1}^{t-1}w_{s}I_{t-s}\text{.}$$

The assumption that the number of cases arising at any timestep is drawn from a Poisson distribution with this mean is common to a number of previous studies in which renewal equation models have been used to quantify pathogen transmissibility from outbreak data (Cori et al., 2013; Nash et al., 2022, 2023; Ogi-Gittins et al., 2024; Thompson, Stockwin, et al., 2019; Thompson et al., 2024). In this expression, ${\left\{I_{k}\right\}}_{k=1}^{t-1}$ denotes the weekly numbers of cases prior to week t and R represents the reproduction number in the absence of the ERT. The variable $w_{s}$ represents the probability that the serial interval (i.e., the period between symptom onset times in an infector-infectee transmission pair) takes the value s weeks (where here we are considering a serial interval distribution that takes values corresponding to positive integer numbers of weeks). The notation w represents the set of values of $w_{s}$ (s=1,2,3,…). In our analyses, we assume that the serial interval distribution is a weekly discretised gamma distribution, where the corresponding continuous time distribution has mean 2.19 weeks (i.e., 15.3 days) and standard deviation 1.3 weeks (i.e., 9.3 days) based on a previous serial interval estimate obtained during the 2014-16 Ebola epidemic in West Africa (WHO Ebola Response Team, 2014). Further details about the procedure used to obtain the discretised serial interval distribution are provided in Text S1.

We estimate the reproduction number in the absence of the ERT, R, via calculation of the likelihood

$$L\left(R\right)=\prod_{t=2}^{T}\frac{{\left(R\sum_{s=1}^{t-1}w_{s}I_{t-s}\right)}^{I_{t}}\;\exp\left(-R\sum_{s=1}^{t-1}w_{s}I_{t-s}\right)}{I_{t}!}\text{,}$$

(1) $$\propto R^{\sum_{t=2}^{T}I_{t}}\;\exp\left(-R\sum_{t=2}^{T}\sum_{s=1}^{t-1}w_{s}I_{t-s}\right).$$

In this expression, week t=1 is assumed to be the epidemiological week from 2nd April to 8th April, and week t=T is assumed to be the epidemiological week from 30th April to 6th May (i.e., the final full week before the arrival of the ERT; in this specific outbreak, T=5). Hence, the value of R is estimated using weekly disease incidence data covering the time period from 2nd April to 6th May.

Adopting a Bayesian approach and assuming an improper prior for R (specifically, assuming that all positive values of R are equally likely *a priori*), then, from equation (1), we note that the posterior for R is a gamma distribution with shape parameter $1+\sum_{t=2}^{T}I_{t}$ and rate parameter $\sum_{t=2}^{T}\sum_{s=1}^{t-1}w_{s}I_{t-s}$. The mean estimate of R is then $\frac{1+\sum_{t=2}^{T}I_{t}}{\sum_{t=2}^{T}\sum_{s=1}^{t-1}w_{s}I_{t-s}}$, and we use this mean estimate of R in our analyses.

In addition to estimating the reproduction number in the absence of the ERT, we also infer the reproduction number under the same transmission model but when the ERT is in place, denoted $R_{ERT}$. This involves the analogous calculations to those described earlier in this subsection, but instead replacing the products and sums from t=2 to t=T=5 with products and sums from t=T+2=7 to t=T+11=16 (these are the epidemiological weeks covering the period from 14th May to 22nd July, which were the full weeks for which the ERT was in place; we note that the sums over the variable s are unchanged when inferring $R_{ERT}$, as cases in week t in this period could have been generated by infectors from any week prior to week t).

### 2.3 The probability of future cases

#### 2.3.1 Perfect reporting

We consider a scenario in which the current week is t. We assume that the incidence data ${\left\{I_{k}\right\}}_{k=1}^{t-1}$ have been observed, and we wish to calculate the probability that cases occur at any time from week t (inclusive) onwards if the ERT is removed immediately (i.e., assuming that virus transmissibility is characterised by the reproduction number in the absence of the ERT, R, estimated as described in section 2.2). We term this quantity the “probability of future cases”, denoted by p(t).

If all cases are reported, then the probability of future cases is

$$p\left(t\right)=1-\mathbb{P}\left(\text{no}\;\text{cases}\;\text{from}\;\text{week}\;t\;\text{onwards}\right)=1-\mathbb{P}\left(I_{t}=0\;|{\;\left\{I_{k}\right\}}_{k=1}^{t-1}\right)\times \prod_{s=t+1}^{\infty }\mathbb{P}\left(I_{s}=0\;|\;{\left\{I_{k}\right\}}_{k=1}^{t-1},\;{\left\{I_{k}=0\right\}}_{k=t}^{s-1}\right)\text{.}$$

Under the transmission model described in section 2.2, this expression can be simplified to obtain

(2) $$p\left(t\right)=1-\exp\left(-R\gamma \left(t\right)\right),$$

where $\gamma \left(t\right)=\sum_{j=t}^{\infty }\sum_{s=1}^{j-1}I_{j-s}w_{s}$, in which the values of $I_{j-s}$ are zero whenever j−s≥t since we are calculating (one minus) the probability that no cases occur from week t onwards (Thompson et al., 2024). For practical purposes, we evaluate the sum over j in the expression for γ(t) from j=t to j=t+50, since the probability of cases being generated following a period of 50 weeks without cases is negligible under our assumed serial interval distribution.

#### 2.3.2 Case under-reporting

While equation (2) provides a useful estimate of the probability of future cases in the absence of the ERT that is straightforward to calculate, case under-reporting is not accounted for. We now extend the approach described in section 2.3.1 to also account for under-reporting.

To do this, we differentiate between the true number of new cases in week t (i.e., $I_{t}$, which includes both reported and unreported cases) and the number of new reported cases in week t, which we denote $C_{t}$. For a given probability that each case is reported, ρ, we use Gibbs sampling to consider a range of plausible values of ${\left\{I_{k}\right\}}_{k=1}^{t-1}$ underlying the observed values of ${\left\{C_{k}\right\}}_{k=1}^{t-1}$. We assume that the number of reported cases in any week, $C_{k}$, is drawn from a binomial distribution with $I_{k}$ trials and reporting probability ρ.

We utilise the following approach, involving Gibbs sampling (Gelfand & Smith, 1990), to estimate the probability of any future cases occurring from time t onwards. For comparability with the result shown in Fig. 2B, we refer to this quantity as the “probability of future cases”, and note that here we are quantifying the probability of any future cases irrespective of whether or not they are reported. First, we set up an initial guess for the values of ${\left\{I_{k}\right\}}_{k=1}^{t-1}$ by scaling up the values of ${\left\{C_{k}\right\}}_{k=1}^{t-1}$ deterministically (to obtain our initial guess for $I_{k}$, we multiply $C_{k}$ by 1/ρ and round to the nearest integer). Then, for j=1,2,…,t−1 in turn, we assume that ${\left\{I_{k}\right\}}_{k=1}^{t-1}$ takes its current guess except for k=j, and replace the current guess for $I_{j}$ with a sample from the probability distribution

(3) $$\mathbb{P}\left(I_{j}\;|\;{\left\{I_{k}\right\}}_{k=1}^{j-1},{\left\{I_{k}\right\}}_{k=j+1}^{t-1}\right)=\left\{ \begin{array}{c} 0,\;\text{for}\;I_{j}<C_{j},\\ \frac{1}{M}\left(\begin{array}{l} I_{j}\\ C_{j} \end{array}\right)\rho^{C_{j}}{\left(1-\rho \right)}^{I_{j}-C_{j}}\prod_{i=2}^{t-1}\frac{{\left(R_{i}\sum_{s=1}^{i-1}w_{s}I_{i-s}\right)}^{I_{i}}\;\exp\left(-R_{i}\sum_{s=1}^{i-1}w_{s}I_{i-s}\right)}{I_{i}!},\;\text{for}\;I_{j}\geq C_{j}. \end{array}\right.$$

This formula represents the probability that there were $I_{j}$ cases in week j, conditional on the number of reported cases in week j and the current guesses for the values of $I_{k}$ in all weeks k≠j. The variable M is a normalising constant (i.e., it is chosen such that $\mathbb{P}\left(I_{j}\;|\;{\left\{I_{k}\right\}}_{k=1}^{j-1},{\left\{I_{k}\right\}}_{k=j+1}^{t-1}\right)$ sums to one over all $I_{j}\geq C_{j}$). In our main analyses, the variable $R_{i}$ is assumed to take the value R (i.e., the reproduction number in the absence of the ERT) in any week i in which the ERT was not in place at all during that week, and $R_{ERT}$ in all other weeks (i.e., any week in which the ERT was in place for part or all of the week).

Having replaced the current guess for all ${\left\{I_{k}\right\}}_{k=1}^{t-1}$, we then repeat this process P times (corresponding to different iterations of the Gibbs sampling procedure; in our main analyses, and except where stated in captions of the relevant supplementary figures, P=100,000) starting from the replaced current guess for ${\left\{I_{k}\right\}}_{k=1}^{t-1}$. We discard the first B iterations as a burn-in and then thin the remaining iterations to retain one in every H iterations (except where stated in captions of the relevant supplementary figures, B=20,000 and H=10). In each of the $\frac{P-B}{H}$ iterations that remain after burn-in and thinning (this corresponds to 8,000 iterations in our main analyses), we compute an estimate of the probability of future cases using equation (2), based on the current guess for ${\left\{I_{k}\right\}}_{k=1}^{t-1}$. We verify convergence of the Gibbs sampler by calculating the Geweke test statistic (Geweke, 1991) from the estimated probability of future cases across all iterations, following removal of the burn-in period and thinning (we check that the p-value corresponding to the calculated Geweke test statistic is always greater than 0.05, as desired). A final estimate of p(t), for a given value of the case reporting probability, ρ, is obtained by calculating the mean of the estimates following the burn-in and thinning.

In addition to estimating the probability of future cases for a fixed value of ρ, we also consider a situation in which there is uncertainty in the value of ρ. In that scenario, we assume that this uncertainty is characterised by a discrete probability distribution governing the value of ρ (i.e., ρ is assumed to take the value $\rho_{i}$ with probability $P\left(\rho =\rho_{i}\right)$, for i=1,2,…,m, say). The probability of future cases is then given by

(4) $$p\left(t\right)=\sum_{i=1}^{m}p\left(t\;|\;\rho =\rho_{i}\right)P\left(\rho =\rho_{i}\right),$$

where $p\left(t\;|\;\rho =\rho_{i}\right)$ is obtained from the Gibbs sampling procedure run for the fixed value of $\rho =\rho_{i}$.

## 3 Results

### 3.1 Comparability of results using weekly and daily incidence data

We began by estimating the value of R based on incidence data from the period from 2nd April to 6th May, to characterise transmission in the absence of the ERT. Not only did we estimate R using the weekly disease incidence data (Fig. 2A – black dashed), which were made available publicly during the outbreak, but we also repeated our inference using the underlying daily disease incidence data (Fig. 2A – blue). We obtained similar mean estimates of R from both the weekly and daily incidence data (2.79 and 2.72, respectively).

We then used the estimated mean values of R to infer the probability of future cases at the beginning of each week, based on disease incidence observed up to the end of the previous week, assuming that all cases were reported. We again compared our results using either the weekly aggregated (Fig. 2B – black dashed) or daily (Fig. 2B – blue) disease incidence data. Similarly to the estimated values of R, we found close correspondence between the estimates, suggesting that collection of daily data is not always required to estimate the probability of future cases precisely.

In theory, policy makers might choose to declare an outbreak over and withdraw the ERT when the estimated probability of future cases falls below a specified threshold (e.g. 0.05 – corresponding to a 5% risk of future cases). In the analysis shown in Fig. 2B, the first week start date on which the estimated probability of future cases fell below 0.05 was 23rd July when either the daily or weekly disease incidence data were used. In reality, the actual ERT withdrawal date was 24th July. If instead an alternative threshold of 0.01 (corresponding to a 1% risk of future cases) was considered, then the first week start date on which the estimated probability of future cases fell below this threshold was 6th August (again using either the daily or weekly disease incidence data).

### 3.2 Accounting for case under-reporting

Having demonstrated that estimates of the probability of future cases are consistent when either daily or weekly incidence data are used, we explored the effect of case under-reporting on the probability of future cases estimated from weekly incidence data (using the Gibbs sampling approach described in section 2.3.2).

Before applying the method to the EVD outbreak dataset, we first tested its reliability using a smaller synthetic dataset for which the probability of future cases could be determined using repeated model simulation (Text S2 and Fig. S2). Specifically, in that supplementary analysis, we found that the Gibbs sampling method provided estimates of the probability of future cases that are indistinguishable by eye from the approach involving repeated model simulation (which would not be feasible for large outbreak datasets).

**Fig. 3:**
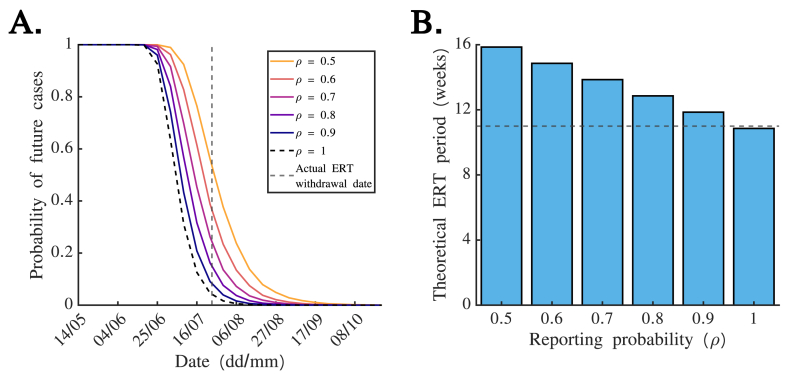
**Estimated probability of future cases accounting for case under-reporting.** A. *Quasi* real-time estimates of the probability of future cases, for different values of the reporting probability ρ. The actual ERT withdrawal date is indicated using a grey dashed vertical line. B. The theoretical period for which the ERT would have been present, if the ERT had been withdrawn as soon as the estimated probability of future cases (as evaluated at the beginning of each epidemiological week) fell below a threshold value of 0.05 (corresponding to a 5% risk of future cases), for different values of the reporting probability ρ. The actual period for which the ERT was in place is denoted by a grey dashed horizontal line.

We then applied the Gibbs sampling method to the EVD outbreak dataset to infer the probability of future cases for a range of different values of the case reporting probability, ρ (Fig. 3A). As might be expected, we found that a higher assumed value of ρ generally led to lower estimates of the probability of future cases, since it then became less likely that recent cases had been missed with the potential to generate additional cases in future. Crucially, we found that this could lead to substantial differences in the theoretical date that the ERT would be withdrawn, if withdrawal occurred as soon as the estimated probability of future cases fell below a specified threshold. For example, if the ERT was withdrawn when the estimated probability of future cases fell below 0.05 (as evaluated at the start of each week), then the ERT withdrawal date would have been 27th August if ρ=0.5 compared to 6th August if ρ=0.8. Different values of ρ therefore corresponded to different theoretical periods for which the ERT would have to be deployed (Fig. 3B).

**Fig. 4:**
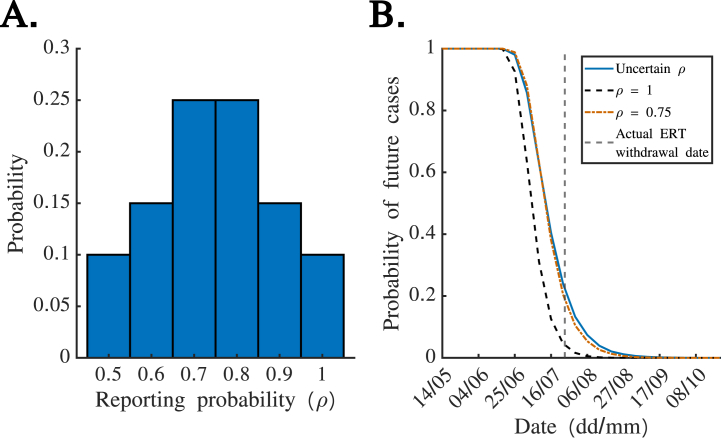
**Accounting for uncertainty in the case reporting probability,**ρ**, when estimating the probability of future cases.** A. Assumed distribution characterising uncertainty in the true value of ρ (approximately reflecting a range of estimated values of ρ at the ends of previous EVD outbreaks (Dalziel et al., 2018)). The probabilities of ρ taking the values {0.5, 0.6, 0.7, 0.8, 0.9, 1} are assumed to be {0.1, 0.15, 0.25, 0.25, 0.15, 0.1}. B. Corresponding *quasi* real-time estimates of the probability of future cases (blue; equation (4)). For comparison, the estimated probability of future cases is also shown under the assumption of perfect case reporting (black dashed; equation (2)) and under the assumption that ρ=0.75 (red dash-dotted; this corresponds to the mean value of the distribution shown in panel A). The actual ERT withdrawal date is indicated using a grey dashed vertical line.

We then considered how uncertainty in the case reporting probability can be built into estimates of the probability of future cases using equation (4). As an example, we assumed that ρ is characterised by the probability distribution shown in Fig. 4A. The corresponding *quasi* real-time estimates of the probability of future cases are shown in Fig. 4B (blue), alongside the analogous estimates in which either perfect reporting (ρ=1) was assumed (Fig. 4B – black dashed) or the mean of the distribution shown in Fig. 4A (ρ=0.75) was assumed (Fig. 4B – red dash-dotted). If the ERT was withdrawn when the estimated probability of future cases fell below 0.05 (as evaluated at the start of each week), then the ERT withdrawal date would have been 13th August if either the distributional estimate of ρ or ρ=0.75 was used. If instead the ERT was withdrawn when the estimated probability of future cases fell below 0.01, then the ERT withdrawal date would have been 3rd September if the distributional estimate of ρ was used, compared to 27th August if instead ρ=0.75 was assumed.

### 3.3 Robustness of results to the modelling assumptions

Finally, we performed two supplementary analyses to evaluate the robustness of our main results to some of the modelling assumptions that we made.

In the transmission model, the number of cases in any week t depends on one of the reproduction numbers, R and $R_{ERT}$; the relevant value is chosen based on whether or not the ERT is in place in week t. In reality, rather than being effective immediately, there may be a delay following the deployment of the ERT during which its control measures have not yet had any effect. The ERT was deployed midway through week t=6. In our first supplementary analysis (described in detail in Text S3), we therefore considered a scenario in which the ERT was assumed not to take effect until week t=8 (so that transmission was governed by R, rather than $R_{ERT}$, up until the beginning of week t=8). The analogous results to Fig. 3 under this alternative assumption are shown in Fig. S3.

In our main analyses, we used values of R and $R_{ERT}$ that were estimated directly from the observed disease incidence time series as described in section 2.2. In doing this, under-reporting was not accounted for when inferring R and $R_{ERT}$. To verify that this did not affect our results, in our second supplementary analysis (described in detail in Text S4) we repeated the analysis shown in Fig. 3 but using values of R and $R_{ERT}$ that were estimated while accounting for under-reporting using Gibbs sampling. The results from this analysis are shown in Fig. S4.

Crucially, in both of our supplementary analyses, our main finding was unchanged: under-reporting necessitates a longer period to have elapsed without reported cases before the ERT can be removed safely (i.e., with only a small chance of further cases occurring).

## 4 Discussion

Towards the end of an infectious disease outbreak, accurate identification of when a pathogen ceases to pose a substantial threat is useful to guide public health decision making. Specifically, when there is a low risk of future cases occurring, policy makers may relax or remove control interventions, reducing the burden on the host population and saving substantial economic costs. For multiple diseases, most notably EVD, the WHO recommends that outbreaks are declared over when two theoretical maximal incubation periods have passed without new cases occurring (for EVD, this corresponds to a period of 42 days (Keita et al., 2022; WHO, 2020)). However, this does not account for specific features of the outbreak in question, such as the level of reporting, which affect the probability that an outbreak is over after a fixed period without cases (Plank et al., 2025; Thompson, Morgan, & Jalava, 2019). As a result, infectious disease modellers have recently been constructing outbreak-specific estimates of the end-of-outbreak probability (or, equivalently, the probability that additional cases will occur in future) using epidemiological data from the specific outbreak under consideration (Akhmetzhanov et al., 2021; Linton et al., 2021, 2022; Nishiura, Miyamatsu, & Mizumoto, 2016; Nishiura et al., 2016; Parag et al., 2020).

Previous quantitative frameworks have been developed for inferring when outbreaks are over that account for case under-reporting. For example, Thompson et al. (Thompson, Morgan, & Jalava, 2019) estimated the end-of-outbreak probability using stochastic simulations of a compartmental model in which infectious individuals either report disease or are cryptically infectious (i.e., do not report disease). However, in that study, end-of-outbreak probability estimates were not based on disease incidence data from any individual outbreak, and therefore outbreak-specific end-of-outbreak probability estimates were not obtained. Djaafara et al. (Djaafara et al., 2021) and Thompson et al. (Thompson et al., 2024) did construct outbreak-specific end-of-outbreak probability estimates, including scenarios that considered case under-reporting, but in those studies the timing of each unreported case was assumed to depend only on the times of reported cases. Instead, the timing of an unreported case should depend on the times at which all other cases occurred, including both reported and unreported cases, as accounted for in our Gibbs sampling approach (see equation (3)). Furthermore, in the study by Djafaara et al. (Djaafara et al., 2021), unreported cases were only considered to have occurred after the final reported case. This could, in principle, affect end-of-outbreak probability estimates. For example, in an outbreak with a large number of unreported cases around the time of the final reported case, there might be a higher probability of future cases compared to in a similar outbreak with a small number of unreported cases around the time of the final reported case.

We have built on this previous literature and obtained two main results. First, for the EVD outbreak considered here, we found that similar estimates of the probability of future cases would have been obtained in real-time based on either daily or weekly disease incidence time series (Fig. 2). Only weekly incidence data were made publicly available during this outbreak; our results suggest that this would not have negatively affected our ability to infer the probability of future cases. Second, we found that the assumed extent of case reporting affects estimates of the probability of future cases (for a given time series describing the previous incidence of reported cases), with a higher assumed case reporting probability corresponding to a lower probability of future cases (Fig. 3). Public health decision makers could theoretically choose to declare an outbreak over when the probability of future cases falls below a pre-specified threshold value. If a public health decision maker prefers to make a risk averse decision, then a lower case reporting probability could be assumed. Alternatively, it is possible to account for uncertainty about the value of the case reporting probability when inferring the probability of future cases using our approach (Fig. 4). This may yield similar or different estimates of the probability of future cases compared to assuming a single value of the case reporting probability.

While our study enables the probability of future cases to be inferred from disease incidence time series data while accounting for under-reporting, our approach involved several simplifying assumptions. For example, we assumed that transmission in the absence of the ERT can be characterised using a single value of the reproduction number, R. In practice, there is uncertainty in the value of R, and this could in principle be accounted for in our approach by using the full distributional estimate of R (Fig. 2A) when calculating the probability of future cases, as in the earlier study by Thompson et al. (Thompson et al., 2024). In such an analysis, it might be important to account for under-reporting when estimating R (as in Fig. S4), since under-reporting can generate uncertainty in estimates of reproduction numbers (Ogi-Gittins et al., 2025). Similarly, our results depend on the assumed serial interval distribution. In practice, this distribution not only varies between pathogens (Vink et al., 2014) but also between outbreaks of the same pathogen (Van Kerkhove et al., 2015) (and even during outbreaks (Ali et al., 2024; Hart et al., 2022)). As such, outbreak-specific serial interval estimates should be used in our approach when they are available, and uncertainty in serial interval estimates could be incorporated into future analyses (Thompson, Stockwin, et al., 2019). Further investigation is required to understand the conditions under which robust inference of the probability of future cases is possible from weekly, rather than daily, disease incidence data. For pathogens with shorter serial intervals than EVD, for example, daily incidence data may be needed for accurate estimation. Additionally, the stochasticity assumed in the renewal equation model underlying our analysis does not account for the possibility of super-spreading events, which could be accounted for by using a negative binomial distribution, rather than Poisson distribution, to model the number of cases each week (Bajaj et al., 2025; Parag, 2021). Finally, other possible extensions of the research presented here include incorporating different modes of transmission (Evans et al., 2025; Lee & Nishiura, 2019) or known heterogeneities between specific groups in the host population (Bolzoni et al., 2007; Bouros et al., 2024; Creswell et al., 2022) into the modelling framework. This latter extension could be particularly important, since often some of the final cases in an outbreak can be in hard-to-reach groups that are associated with different characteristics to the majority of the population (Klepac et al., 2015).

Despite these simplifications, we have shown that the accuracy of real-time estimates of the probability of future cases at the tail end of an infectious disease outbreak may not always be improved by collecting finer temporal resolution disease incidence data, and we have provided an epidemiological modelling framework for inferring the probability of future cases while accounting for case under-reporting. Since under-reporting occurs during outbreaks of most diseases (Ducrot et al., 2022; Gibbons et al., 2014; Liu et al., 2020), and – as we have shown – affects model-derived conclusions about when outbreaks can be declared over with only a limited risk of future cases, we contend that under-reporting should always be considered when deciding when to declare an outbreak over. As Professor Pierre Magal and coauthors stated in their research undertaken in the first few months of the COVID-19 pandemic (Griette et al., 2022), “the end date of an epidemic wave depends sensitively on the proportion of infectious cases that report disease”. We hope that the approach presented here provides a quantitative framework that can be used to inform decision making after the apparent ends of infectious disease outbreaks.

## CRediT authorship contribution statement

**I. Ogi-Gittins:** Writing – review & editing, Writing – original draft, Visualization, Validation, Methodology, Investigation, Formal analysis. **J. Polonsky:** Writing – review & editing, Investigation, Data curation. **M. Keita:** Writing – review & editing, Investigation, Data curation. **S. Ahuka-Mundeke:** Writing – review & editing, Investigation, Data curation. **W.S. Hart:** Writing – review & editing, Methodology. **M.J. Plank:** Writing – review & editing, Methodology. **B. Lambert:** Writing – review & editing, Methodology. **E.M. Hill:** Writing – review & editing, Writing – original draft, Validation, Supervision. **R.N. Thompson:** Writing – review & editing, Writing – original draft, Supervision, Project administration, Methodology, Conceptualization.

## Data availability

The computing code used to perform the analyses in this article is available, along with relevant data, in the following GitHub repository: https://github.com/billigitt/End_of_Outbreak_Probs_Underrep/. All code was written in MATLAB (compatible with version 2021b).

## Funding

This research was funded by the 10.13039/501100000266EPSRC through the Mathematics for Real-World Systems CDT (EP/S022244/1; IOG). The authors acknowledge the help and support of the JUNIPER Consortium (MR/X018598/1; EMH, RNT). EMH is affiliated to the National Institute for Health and Care Research Health Protection Research Unit (NIHR HPRU) in Gastrointestinal Infections at the University of Liverpool (PB-PG-NIHR-200910), in partnership with the UK Health Security Agency (UKHSA) and in collaboration with the University of Warwick (EMH is based at The University of Liverpool). This research was also funded by The Pandemic Institute, formed of seven founding partners: 10.13039/501100000836The University of Liverpool, 10.13039/100014976Liverpool School of Tropical Medicine, 10.13039/501100004144Liverpool John Moores University, 10.13039/501100019150Liverpool City Council, Liverpool City Region Combined Authority, Liverpool 10.13039/501100018911University Hospital Foundation Trust and Knowledge Quarter Liverpool (as above, EMH is based at 10.13039/501100000836The University of Liverpool). The views expressed are those of the authors and not necessarily those of the NIHR, the Department of Health and Social Care, the UKHSA or The Pandemic Institute. For the purpose of Open Access, the authors have applied a CC BY public copyright licence to any Author Accepted Manuscript (AAM) version arising from this submission.

## Declaration of competing interest

The authors declare that they have no known competing financial interests or personal relationships that could have appeared to influence the work reported in this paper.
