# Supplementary material for: Real-time inference of the end of an outbreak: Temporally aggregated disease incidence data and under-reporting
Source: Infect Dis Model. 2025 Apr 1;10(3):935–45. doi: 10.1016/j.idm.2025.03.009 (PMC12138552; doi:10.1016/j.idm.2025.03.009)
Supplement: Multimedia component 1 [file mmc1.pdf]

Supplementary Information for *Real-time inference of the end of an outbreak: Temporally aggregated disease incidence data and under-reporting*

I Ogi-Gittins, J Polonsky, M Keita, S Ahuka-Mundeke, WS Hart, B Lambert, MJ Plank, EM Hill, RN Thompson

Supplementary Text

Text S1. Serial interval discretisation

A key input to our analyses is the discrete probability distribution characterising the serial interval (i.e., the period between symptom onset times in infector-infectee transmission pairs). A serial interval distribution was estimated for EVD during the 2014-16 epidemic in West Africa [1]. Specifically, the authors of that study estimated the parameters of a (continuous) gamma distribution, obtaining a serial interval distribution with mean 15.3 days (i.e., 2.19 weeks) and standard deviation 9.3 days (i.e., 1.3 weeks). To discretise this distribution into weekly values, we follow the approach described by Cori *et al.* [2] and Ogi-Gittins *et al.* [3] and calculate

$$w_k = \int_0^\infty \mathbb{P}(\text{discrete SI} = k \mid \text{continuous SI} = u) \times g(u)\mathrm{d}u,$$

$$= \int_{k-1}^{k+1} (1 - |u - k|)g(u)\,\mathrm{d}u,$$

for $k = 2,3, \dots$ weeks, in which $g(u)$ is the probability density at value u of a gamma distribution with mean 2.19 weeks and standard deviation 1.3 weeks. The value of w_1 is then chosen so that the set of values of w_k (denoted $\boldsymbol{w}$ in the main text) represents a valid probability distribution (i.e., $\sum_{k=1}^{\infty} w_k = 1$).

When we apply our approach to daily incidence data (in Fig 2 of the main text), we use the same approach to discretise the serial interval distribution but instead discretise it into daily values (performing the same calculation, but where $g(u)$ is the probability density at value u of a gamma distribution with mean 15.3 days and standard deviation 9.3 days).

Text S2. Testing the Gibbs sampling approach

We tested the Gibbs sampling method for inferring the probability of future cases using a simple example in which this probability could also be estimated using repeated model simulations. While the simulation-based method is straightforward to use, estimates require substantial computational resources for even relatively small disease incidence time series datasets, prompting our decision to use Gibbs sampling for the EVD outbreak dataset analysed in the main text.

We consider a synthetic outbreak "dataset" in which two cases were reported, with one reported case in each of weeks $t = 1$ and $t = 3$. We then undertake quasi real-time inference each week of the probability of future cases, from the beginning of week $t = 4$ onwards. To estimate the probability of future cases at the beginning of week t using the simulation-based method, we repeatedly simulate the renewal equation model described in section 2.2 of the main text. Specifically, in each simulation we: i) Sample the number of cases in week 1 (I_1), based on the observation of a single reported case in that week ($C_1 = 1$), from a (normalised) binomial likelihood with I_1 trials, reporting probability $\rho = 0.5$ and $C_1 = 1$ reported case*; ii) Simulate the numbers of cases in subsequent weeks ($I_2, I_3, I_4 \dots$) using the renewal equation model, assuming that the reproduction number $R = 0.5$ and the ERT is never deployed; and

*In step i of the simulation-based method, we sample the value of I_1 from the probability distribution

$$\mathbb{P}(I_1 \text{ infected individuals in week 1}) = \frac{1}{N_1}\binom{I_1}{1}\rho(1-\rho)^{I_1-1}, \qquad \text{for } I_1 = 1,2,3,\dots$$

in which N_1 is a normalising constant so that this represents a valid probability distribution.

iii) Sample the number of reported cases in week k (i.e., C_k), for $k \geq 2$, from a binomial distribution with I_k trials and reporting probability $\rho = 0.5$. We continue this procedure until 200,000 simulations have been generated in which the simulated incidence of reported cases matches the synthetic outbreak dataset exactly in the time period up to and including week $t - 1$. We then calculate the proportion of those matching simulations in which cases occur after week t (inclusive), providing an estimate of the probability of future cases that is conditioned to the synthetic dataset.

The outputs of the simulation-based method and the Gibbs sampling approach (again under the assumptions that $R = 0.5$, $\rho = 0.5$ and the ERT is never deployed) for this small synthetic dataset are shown in Fig S2. The results from the two methods are identical (at least by eye), indicating that the Gibbs sampling method provides consistent estimates of the probability of future cases for this dataset.

Text S3. Accounting for delayed effectiveness of the ERT

As described in the main text, in reality the ERT was deployed midway through week $t = 6$. In our main analyses, we estimated R (in the absence of the ERT) based on reported cases occurring up to and including week $t = T = 5$, and estimated R_{ERT} based on observed case numbers from week $t = T + 2 = 7$ onwards. When we inferred the total number of cases each week while accounting for under-reporting (as described in section 2.3.2 of the main text; Figs 3 and 4), we made the additional assumption that transmission in week $t = 6$ was governed by R_{ERT}.

In practice, the ERT is unlikely to be effective immediately. Instead, there may be a delay while the measures put in place by the ERT are implemented. For that reason, here we perform a supplementary analysis in which we repeat the results shown in Fig 3 of the main

text, but instead assuming that case numbers observed up to week $t = 7$ (inclusive) are determined by the pre-ERT reproduction number, R, and that case numbers observed from week $t = 8$ onwards are determined by R_{ERT}. In this supplementary analysis, we re-infer R and R_{ERT} as described in section 2.2 of the main text, and account for under-reporting as described in section 2.3.2 of the main text, but assuming throughout that the ERT only becomes effective from week $t = 8$ onwards.

The results of this supplementary analysis are shown in Fig S3. While the precise estimates of the probability of future cases are not identical between Figs 3 and S3, our main conclusion is unchanged. Under-reporting necessitates waiting for a longer period without reported cases before an outbreak can be declared over confidently.

Text S4. Accounting for under-reporting when estimating reproduction numbers

In the analyses presented in the main text, we estimated R and R_{ERT} directly from the observed disease incidence time series without accounting for under-reporting (see section 2.2 of the main text). To verify the robustness of our results, here we repeat the analysis presented in Fig 3 of the main text, but instead accounting for under-reporting when estimating R and R_{ERT}. To do this, we use Gibbs sampling.

First, we set up an initial guess for the values of I_k (for $k = 1,2, \dots 16$) by scaling up the values of C_k deterministically (multiplying C_k by $\frac{1}{\rho}$ and rounding to the nearest integer).

We then repeat the following two steps for $j = 1,2, \dots 16$: i) We calculate the posterior distributions for R and R_{ERT} as described in section 2.2 of the main text but using the current guess for the values of $\{I_k\}_{k=1}^{16}$, and sample individual values of R and R_{ERT} from those posteriors; ii) Using those sampled values of R and R_{ERT}, and the current guess for all I_k

except I_j, we calculate $\mathbb{P}\left(I_j \text{ cases in week } j \mid \{I_k\}_{k=1}^{j-1}, \{I_k\}_{k=j+1}^{16}\right)$ using equation (3) in the main text, and sample a value from that distribution to become the new guess for I_j. This process is then repeated for $100{,}000$ iterations, and the sampled values of R and R_{ERT} are stored after each iteration (i.e, after each calculation undertaken when $j = 16$).

We implement a burn-in (of 10,000 iterations) and thinning (retaining one in every 10 iterations), giving 9,000 possible values of each of R and R_{ERT}. We verify convergence of the Gibbs sampler, again by checking that the p-value corresponding to the Geweke test statistic exceeds 0.05. We calculate the mean of the 9,000 possible values of R and the mean of the 9,000 possible values of R_{ERT}. We then use those mean estimates as inputs to calculate the probability of future cases using the method described in section 2.3.2 of the main text (i.e., accounting for under-reporting using a further Gibbs sampling procedure). The analogous results to Fig 3 in the main text, but accounting for under-reporting when estimating R and R_{ERT} as described here, are shown in Fig S4.

The results shown in Fig S4 are similar to those shown in Fig 3 of the main text. Crucially, our main conclusion is again unchanged. In the presence of under-reporting, it is necessary to wait for a longer period without reported cases before the ERT can be withdrawn with only a small chance of future cases.

Supplementary Figures

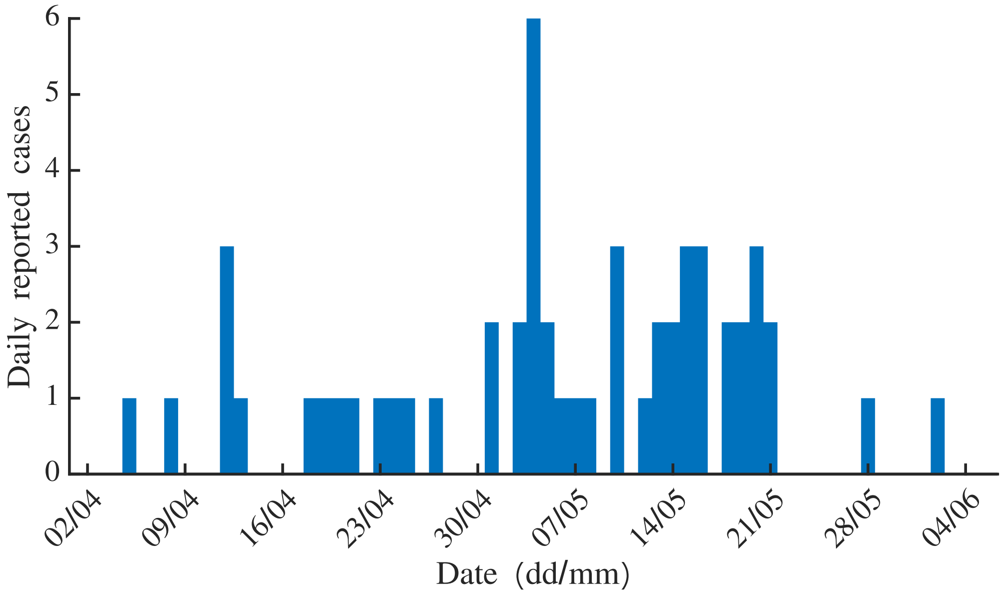

Figure S1. Daily numbers of reported cases in the 2018 EVD outbreak in Équateur Province, DRC. In total, 54 cases occurred between 5th April and 2nd June (inclusive).

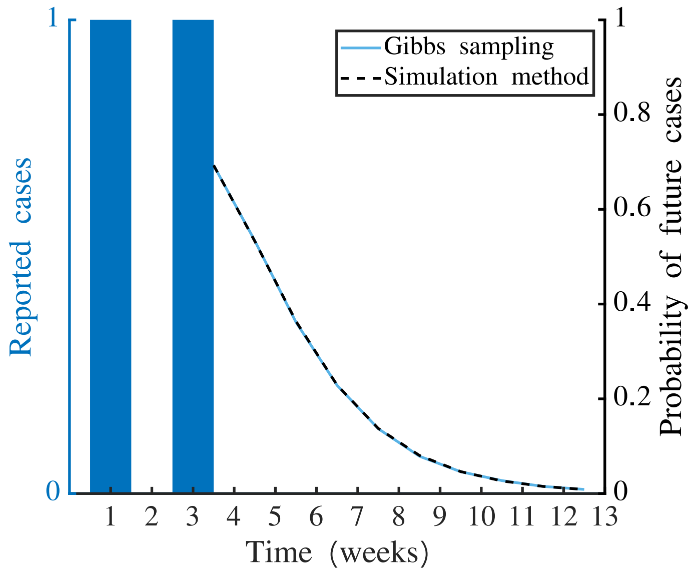

Figure S2. Comparison of results from the Gibbs sampling method (as used in the main text) and an alternative approach involving repeated model simulation. The synthetic dataset, comprised of single cases in weeks one and three, is represented by the blue bars. Real-time estimates of the probability of future cases obtained at the beginning of each week are shown, for the Gibbs sampling method used in the main text (blue) and for the simulation-based method described in Text S2 (black dashed), under the assumption that the

reproduction number is $R = 0.5$, the reporting probability is $\rho = 0.5$, and the ERT was not deployed in this outbreak. Both methods generate identical results.

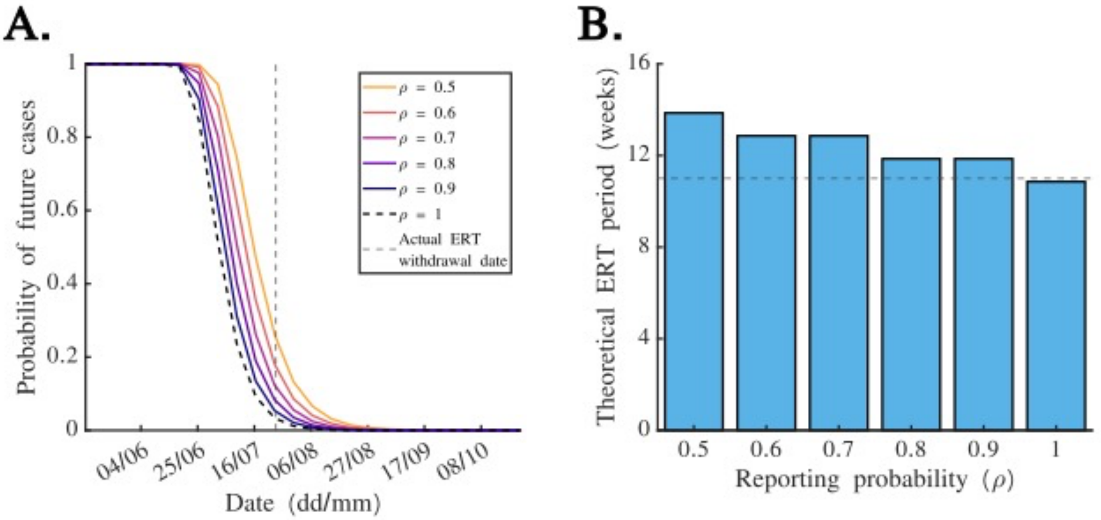

Figure S3. Estimated probability of future cases accounting for a delay between the introduction of the ERT and its measures becoming effective. Analogous results to Fig 3 in the main text, but instead assuming that transmission is only governed by R_{ERT} from week $t = 8$ onwards. For reference, the ERT was deployed during week $t = 6$. In this analysis, when we used Gibbs sampling to account for under-reporting when estimating the probability of future cases, we ran the Gibbs sampler for a longer period than in the analyses in the main text to ensure convergence (as verified by checking that the p-value corresponding to the Geweke test statistic exceeded 0.05). Specifically, we used sampling parameter values of $P = 1{,}000{,}000$, $B = 300{,}000$ and $H = 10$.

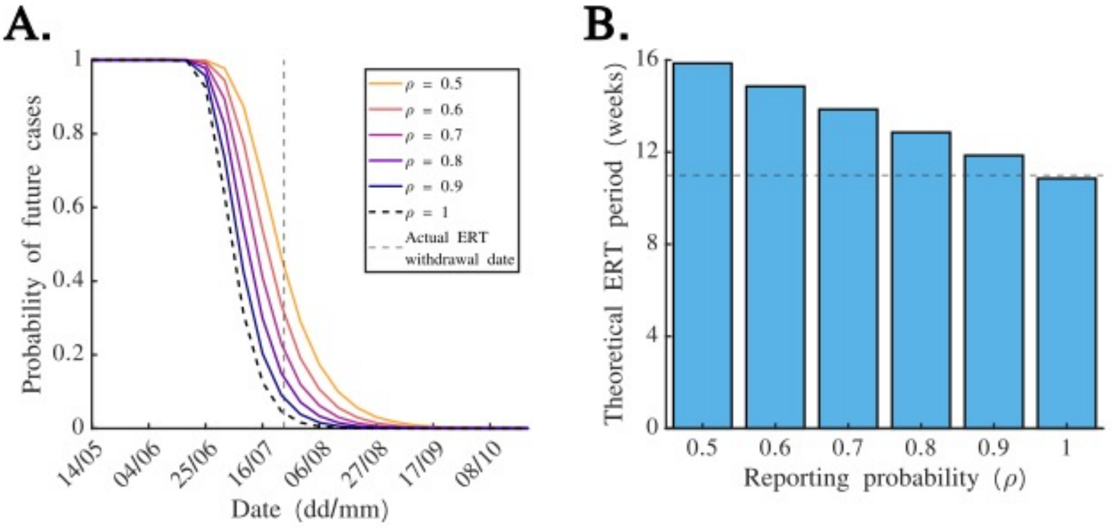

Figure S4. Estimated probability of future cases accounting for under-reporting when estimating R and R_{ERT}. Analogous results to Fig 3 in the main text, but accounting for under-reporting using Gibbs sampling

when estimating R and R_{ERT} as described in Text S4. In this analysis, after estimating R and R_{ERT}, when we used Gibbs sampling to account for under-reporting when estimating the probability of future cases, we ran the Gibbs sampler for a longer period than in the main text to ensure convergence (as verified by checking that the p-value corresponding to the Geweke test statistic exceeded 0.05). Specifically, we used sampling parameter values of $P = 1{,}000{,}000, B = 300{,}000$ and $H = 10$.

References

1. WHO Ebola Response Team. Ebola virus disease in west Africa — The first 9 months of the epidemic and forward projections. N Engl J Med. 2014;371: 1481–1495.

2. Cori A, Ferguson NM, Fraser C, Cauchemez S. A new framework and software to estimate time-varying reproduction numbers during epidemics. Am J Epidemiol. 2013;178: 1505–12.

3. Ogi-Gittins I, Hart WS, Song J, Nash RK, Polonsky J, Cori A, et al. A simulation-based approach for estimating the time-dependent reproduction number from temporally aggregated disease incidence time series data. Epidemics. 2024;47: 100773.
